# Survival advantage of cytoreductive surgery and hyperthermic intraperitoneal chemotherapy (HIPEC) for advanced gastric cancer: experience from a Western tertiary referral center

**DOI:** 10.1007/s00423-021-02137-5

**Published:** 2021-02-21

**Authors:** Fausto Rosa, Federica Galiandro, Riccardo Ricci, Dario Di Miceli, Fabio Longo, Giuseppe Quero, Antonio Pio Tortorelli, Sergio Alfieri

**Affiliations:** 1 Digestive Surgery, Fondazione Policlinico Universitario Agostino Gemelli IRCCS, Largo A. Gemelli, 8, 00168 Rome, Italy; 2 Università Cattolica del Sacro Cuore, Rome, Italy; 3 Pathology, Fondazione Policlinico Universitario Agostino Gemelli IRCCS, Rome, Italy; 4 General Surgery, Ospedale Buccheri-La Ferla, Palermo, Italy

**Keywords:** Gastric cancer, HIPEC, Peritoneal carcinomatosis, Surgery

## Abstract

**Background:**

Selection criteria and prognostic factors for patients with advanced gastric cancer (AGC) undergoing cytoreductive surgery (CRS) *plus* hyperthermic intra-operative peritoneal chemotherapy (HIPEC) have not been well defined and the literature data are not homogeneous. The aim of this study was to compare prognostic factors influencing overall (OS) and disease-free survival (DFS) in a population of patients affected by AGC with surgery alone and surgery *plus* HIPEC, both with curative (PCI, Peritoneal Carcinomatosis Index >1) and prophylactic (PCI=0) intent.

**Methods:**

A retrospective analysis of a prospectively collected database was conducted in patients affected by AGC from January 2006 to December 2015. Uni- and multivariate analyses of prognostic factors were performed.

**Results:**

A total of 85 patients with AGC were analyzed. Five-year OS for surgery alone, CRS *plus* curative HIPEC, and surgery *plus* prophylactic HIPEC groups was 9%, 27%, and 33%, respectively. Statistical significance was reached comparing both prophylactic HIPEC vs surgery alone group (*p* = 0.05), curative HIPEC vs surgery alone group (*p* = 0.03), and curative vs prophylactic HIPEC (*p* = 0.04).

Five-year DFS for surgery alone, CRS + curative HIPEC, and surgery + prophylactic HIPEC groups was 9%, 20%, and 30%, respectively. Statistical significance was reached comparing both prophylactic HIPEC vs surgery alone group (*p* < 0.0001), curative HIPEC vs surgery alone group (*p* = 0.008), and curative vs prophylactic HIPEC (*p* = 0.05).

**Conclusions:**

Patients with AGC undergoing surgery *plus* HIPEC had a better OS and DFS with respect to patients treated with surgery alone.

## Introduction

Gastric cancer is the sixth most prevalent malignant tumor worldwide and the third leading cause of cancer-related death. The International Agency for Research on Cancer estimated that there were about one million new cases of gastric cancer and 782.685 deaths from gastric cancer in 2018 [1]. Many patients in the Western world with AGC die from metastases [2].

The peritoneal cavity is also a frequent site for metastatic disease after resection, particularly in patients with serosa-infiltrating tumors [3, 4]. Patients with AGC and peritoneal carcinomatosis (PC) have a poor prognosis, with a median survival of 3.1 months without treatment [5]. Systemic chemotherapy extended the median survival time to 11 months in patients with AGC compared with best supportive care alone [6].

Extended resection involving gastrectomy and peritonectomy combined with administration of HIPEC may improve survival in patients with PC [7–9]. HIPEC possesses a theoretical advantage over systemic treatment delivering high drug concentrations directly to the peritoneal cavity, resulting in a reduced systemic toxicity [10–12]. In addition, high drug concentrations are achieved in the portal vein [13, 14].

Extended survival with HIPEC in AGC has been demonstrated, but the lack of standardized protocols has led to difficulties comparing and interpreting results [15]. A meta-analysis demonstrated improved overall survival with HIPEC with or without early postoperative intraperitoneal chemotherapy [16].

Perhaps the most appropriate use of HIPEC in AGC would be prophylactic, suggesting an adjunct to curative surgical resection in patients with a high risk of peritoneal recurrence. Not surprisingly, the majority of data related to HIPEC in AGC is prophylactic against peritoneal recurrences. The theoretical rationale and synergistic effect is that large diluent volumes in HIPEC wash out most of the intraperitoneal free cancer cells, and chemotherapy destroys remaining cancer cells [17].

With the aim of contributing to this issue, we have conducted a comparative observational analysis between patients undergoing CRS alone and those who received gastrectomy *plus* HIPEC both with curative (PCI >1) and prophylactic (PCI=0) intent.

## Methods

A retrospective analysis of prospectively collected data was conducted regarding patients with AGC observed and treated at Digestive Surgery Unit, Fondazione Policlinico Universitario “A. Gemelli” IRCCS, from January 2006 to December 2015.

We preliminarily obtained Institutional Review Board approval to use patient data.

Patients analyzed were divided into the following 3 groups:

- Surgery *plus* HIPEC with curative intent: AGC patients with apparent peritoneal dissemination who underwent cytoreductive surgery, including gastrectomy and partial peritonectomy of peritoneal sections affected by implants, followed by HIPEC
- Surgery *plus* HIPEC with prophylactic intent: AGC patients with serosa invasion and consequent high-risk of intraperitoneal progression, who underwent gastrectomy followed by HIPEC
- Surgery alone: AGC patients who underwent only gastrectomy due to the presence of exclusion criteria for HIPEC

The same team of oncologists performed all surgeries, and all patients had to provide a written informed consent before the intervention.

Patients were divided according to the type of surgical procedure performed.

### Inclusion/exclusion criteria

All patients were submitted to a complete clinical evaluation, including laboratory tests, with complete blood cell count and serum chemistry.

In order to exclude extra-abdominal disease and to assess the possibility of optimal cytoreduction, all patients underwent to a CT scan or FDG-PET/CT scan. A preoperative laparoscopy was selectively performed for the purpose of selecting patients for neoadjuvant therapy.

Patients with histologically documented AGC, with a preoperative stage II to IV, with peritoneal carcinomatosis (stage IV), or at high risk to develop it due to serosal involvement were included in the study.

Inclusion criteria were as follows: age 18–80 years; normal cardiac, respiratory, liver, and renal functions; and no hematological alterations.

Exclusion criteria for HIPEC were uncontrolled severe infection and/or medical problems unrelated to malignancy which would limit full compliance with the protocol or expose the patient to extreme risk of life.

All patients in surgery alone group were excluded from HIPEC due to the presence of an exclusion criteria.

All patients included were analyzed without defining any cut-off value for PCI and CC score.

We recorded hospital morbidity and mortality, type of treatment, histologic type according to Lauren [18], and demographic characteristics, tumor size, and tumor location. The disease was staged according to the 8^th^ Edition of the American Joint Committee on Cancer and the International Union Against Cancer Staging System (UICC) [19, 20].

### Surgical rules

Based on categories established by the Japanese Gastric Cancer Association [21], the regional extent of nodal involvement after radical procedures was also recorded.

At the end of the operation, the surgeon resected all lymph nodes from the surgical specimen and identified their distribution and tumor location according to the classification by the Japanese Gastric Cancer Association [21].

The PCI score was calculated at laparotomy [22]. The CC score was calculated for all patients in the three groups. CC-0 reflected no remaining visible disease. CC-1, 2, and 3 implied remaining disease less than 2.5 mm, 2.5 to 2.5 cm, and greater than 2.5 cm [22].

After total gastrectomy with D2 lymph node dissection, esophagojejunostomy (using a circular stapler, diameter 25 mm) was used routinely for Roux-en-Y reconstruction.

In case of subtotal gastrectomy, intestinal continuity was restored by means of Billroth II or Roux-en-Y gastrojejunostomy, at discretion of the surgeon.

In case of carcinomatosis, CRS was performed removing all peritoneum and visceral organs involved.

Extensive surgery (associated resections) because of suspicion of direct tumor invasion or carcinomatosis was defined as combined resection of adjacent organs (spleen, left pancreas, liver, colon, adrenal gland, diaphragm, abdominal wall, and small intestine).

### HIPEC

HIPEC was carried out according to the Coliseum technique [22]. Two inflow and two outflow 29 French catheters were placed in the upper and lower abdominal quadrants, respectively. The HIPEC procedure was administered for 90 min with an inflow temperature of 41–42°C and an outflow temperature of 39–40°C, using mitomycin C (MMC) at a dose of 15 mg/m^2^ and cisplatin at a dose of 75 mg/m^2^. As perfusate volume, a 2 L/m^2^ 0.9% NaCl solution was used. At the end of the procedure, an abdominal washout was performed with 3 L of crystalloid solution. After 90 min of perfusion, the abdomen was cautiously re-explored to control the hemostasis.

The temperature was monitored using digital probes placed in abdominal cavity at circuit level.

### Pathological data

Based on definitive pathologic findings, the potentially curative operations were classified as radical (R0-microscopic tumor free) or as R1—microscopic residual disease—according to the presence or absence of residual tumor. Palliative resection was classified based on R2 macroscopic disease left behind. Frozen sections were not routinely used in the evaluation of margins, but only in the suspicion of a possible tumor infiltration.

### Postoperative course

The patients were monitored for 30-day postoperative complications and mortality.

Early postoperative complications were considered occurring within 30 days from surgery and with a severity grade 2 or more according to the Clavien-Dindo classification [23]. All postoperative complications were registered in the database during hospitalization or at the first follow-up, by telephone contact, within 30 days from surgery.

Postoperative mortality was defined as death within 30 days from surgery.

Perioperative chemotherapy was administered, in the majority of cases, according to the MRC Adjuvant Gastric Infusional Chemotherapy (MAGIC) protocol [24].

The oncologists decided about adjuvant chemotherapy administration, as previously reported [25], resulting in heterogeneity regarding chemotherapy, treatment protocols, and a number of cycles performed.

All patients included in the study were regularly followed up with a standardized protocol [26].

### Statistical analyses

All clinical and pathological data were prospectively stored in a GC database and evaluated for this study. All variables are expressed as the mean ± standard deviation (±), median and interquartile range (IQR) when appropriate. The statistical significance of the difference between mean values was evaluated using the Student’s *t*-test. All tests were two tailed. Categorical variables were assessed by the Pearson’s chi-square test. Multivariable analysis was undertaken using the Cox proportional hazards model. The survival adjusted for censoring was calculated using the Kaplan-Meier method, and the medians were compared using the log-rank test. A *p* value <0.05 was considered statistically significant.

All data were analyzed by SPSS version 25® (IBM, IL, USA).

## Results

During the study period, a total of 427 patients with GC underwent surgery with curative intent at the Digestive Surgery Unit of the Fondazione Policlinico Universitario “A. Gemelli” IRCCS of Rome.

Among them, 85 patients with advanced GC were retrospectively analyzed for this observational study. More specifically, forty-six patients (F/M ratio 25/21; mean age 55 years, range 28–76) underwent surgery *plus* HIPEC. In 50% (23/46) of cases, indication for HIPEC was a T3/T4 gastric cancer without peritoneal carcinomatosis (PCI = 0). Thirty-nine patients received CRS alone.

Clinico-demographic characteristics of all patients are shown in Table 1.

**Table 1:** Clinico-demographic characteristics of all patients

Patients, *n*	85
Age, years, mean (± sd)	61 ± 15.1
Female, *n* (%)	44 (52)
Primary tumor location	
Lower third, *n* (%)	36 (42)
Middle third, *n* (%)	34 (40)
Upper third, *n* (%)	15 (18)
Neoadjuvant therapy	
Chemotherapy, *n* (%)	38 (44.7)
No. of cycles, mean (+ sd)	5 ± 4.8
Chemoradiotherapy, *n* (%)	2 (2)
Response to treatment, *n* (%)	19 (50)
ASA, *n* (%)	
1	18 (21)
2	50 (59)
3	17 (20)
Indication for HIPEC	
Prophylactic (PCI=0), *n* (%)	23 (27)
Curative (PCI ≥ 1), *n* (%)	23 (27)
No HIPEC, *n* (%)	39 (46)
Total harvested lymph nodes	
*n*<15 (%)	8 (9.4)
*n*≥15 (%)	77 (90.6)
Positive lymph nodes	
N0, (%)	10 (11.8)
N+, (%)	75 (88.2)
Adjuvant therapy	41 (48.2)
Length of stay, days, mean (+ sd)	13.4 + 9.3
Operation time, minutes, mean (+ sd)	338 + 92.7
Follow-up, months, median (IQR)	68

The median follow-up (IQR) was 68 months.

Excluding 4 patients lost during the study period and 3 patients who died during the postoperative hospital stay (1 in the curative HIPEC group and 2 in the only surgery group), follow-up was completed in 78 cases (91.7%). At the last evaluation, 54 (63.5%) patients had died.

Positive cytology was present only in 6 patients (26%) who underwent prophylactic HIPEC.

Thirty-eight patients (44.7%) received neoadjuvant chemotherapy with a pathological response in 19 cases (50%).

The majority of patients were preoperatively classified as ASA 2 (50 patients, 59%).

Seventy-seven patients (90.6%) had ≥ 15 lymph nodes retrieved and 75 (88.2%) were N+.

The mean duration of surgical procedures was 338 (±92.7) minutes, and the mean length of postoperative hospital stay was 13.4 (±9.3) days.

Forty-one patients (48.2%) received adjuvant chemotherapy.

Clinico-demographic characteristics of the three groups are shown in Table 2.

**Table 2:** Clinico-demographic characteristics of the three study groups

	Prophylactic HIPEC (*n*=23)	Curative HIPEC (*n*=23)	No HIPEC (*n*=39)	p*
Age, years, mean (+)	58 (35–74)	52 (28–76)	68 (41–86)	<0.0001
Sex, *n* (%)				
Male	11 (48)	10 (43)	20 (51)	0.83
Female	12 (52)	13 (57)	19 (49)	
ASA score, *n* (%)				
ASA I	4 (17)	10 (43)	2 (5.1)	0.04
ASA II	14 (61)	10 (43)	12 (30.8)	
ASA III	5 (22)	3 (13)	25 (64.1)	
Tumor location, *n* (%)				
Lower third	8 (35)	6 (26)	22 (56)	0.08
Middle third	12 (52)	10 (43)	12 (31)	
Upper third	3 (13)	7 (31)	5 (13)	
Neoadjuvant therapy	12 (52)	11 (47.8)	15 (38.5)	0.46
pTNM stage, *n* (%)				
IIB	3 (13)	0	4 (10.3)	0.008
IIIA	7 (30)	0	11 (28.2)	
IIIB	2 (9)	0	10 (25.6)	
IIIC	11 (48)	0	4 (10.3)	
IV	0	23 (100)	10 (25.6)	
PCI** range, *n* (%)				
0	23 (100)	0	29 (74.4)	<0.0001
1–6	0	14 (61)	4 (10.2)	
7–15	0	7 (30)	6 (15.4)	
16–39	0	2 (9)	0	
CC score***, *n* (%)				
CC 0	23 (100)	19 (82.6)	32 (82.1)	0.003
CC 1	0	4 (17.4)	2 (5.1)	
CC 2	0	0	2 (5.1)	
CC 3	0	0	3 (7.7)	
R status, *n* (%)				
R0	22 (96)	18 (78.3)	32 (82.1)	0.03
R1	1 (4)	5 (21.7)	2 (5.1)	
R2	0	0	5 (12.8)	
Lauren classification, *n* (%)				
Diffuse type	13 (57)	12 (52)	19 (49)	0.98
Intestinal type	6 (26)	7 (30)	13 (33)	
Mixed type	4 (17)	4 (18)	7 (18)	
Total harvested lymph nodes				
*n*<15 (%)	1 (4)	2 (8)	5 (12)	0.53
*n*>15 (%)	22 (96)	21 (92)	34 (88)	
Positive lymph nodes				
N0, (%)	1 (4)	3 (13)	6 (15)	0.41
N+, (%)	22 (96)	20 (87)	33 (85)	
Adjuvant therapy	18 (78.3)	15 (65.2)	8 (20.5)	0.52

*Two-tailed Pearson’s chi-square test

**PCI Peritoneal Carcinomatosis Index

***CC score cytoreduction completeness score

A significant difference among the three groups was noticed regarding the distribution of ASA score, tumor location and tumor stage, PCI range, CC score, and R status.

Intra-operative and short-term outcomes for the three groups are shown in Table 3.

**Table 3:** Intra-operative and short-term outcomes

	Prophylactic HIPEC (*n*=23)	Curative HIPEC (*n*=23)	No HIPEC (*n*=39)	p*
Type of resection				
Total gastrectomy, *n* (%)	12 (52)	16 (69)	18 (46)	0.19
Subtotal gastrectomy, *n* (%)	11 (48)	7 (31)	21 (54)	
Associated resections, *n* (%)	8 (35)	15 (65)	10 (25)	0.008
Operation time, min, mean (±SD)	380 + 35.6	482 + 42.1	227 + 28.7	<0.0001
EBL^**^, ml, mean (±SD)	204 ± 103.1	250 ± 153.0	190 ± 80.2	0.23
Postoperative complications, *n* (%)	9 (39)	9 (39)	18 (46)	0.8
Surgical complications, *n* (%)	4 (17)	7 (30)	7 (18)	0.44
Evisceration	0	1 (4)	1 (2)	
Intra-abdominal abscess	2 (8)	0	2 (5)	
Anastomotic leakage	1 (4)	1 (4)	4 (10)	
Bowel obstruction	0	1 (4)	0	
Bleeding	0	1 (4)	0	
Delayed gastric emptying	0	1 (4)	0	
Intestinal ischemia	0	1 (4)	0	
Wound infection	1 (4)	1 (4)	0	
Medical complications, *n* (%)^†^	6 (26)	4 (17)	15 (38)	0.19
Clavien-Dindo ≥2, *n* (%)	3 (13)	6 (26)	9 (23)	0.51
Reoperation, *n* (%)	2 (8)	5 (21)	3 (7)	0.21
Length of stay, days, mean (+ sd)	11 ± 5.2	16 ± 3.7	16 ± 4.1	0.06
Postoperative mortality^χ^, *n* (%)	0 (0)	1 (4)	2 (5)	0.55

*Two-tailed Pearson’s chi-square test

^**^EBL estimated blood loss

^**†**^Fever without signs of infection or need of antibiotics, hypertension, electrolyte imbalance, pulmonary atelectasis requiring physiotherapy, transient confusion not requiring therapy

^χ^Death within 30 days from surgery

Among the three groups, a significant difference was detected as far as associated resections and operation time were concerned (*p*=0.008 and *p*<0.0001, respectively).

No differences between the three groups neither in terms of postoperative complications (*p*=0.8) nor in terms of postoperative mortality (*p*=0.55) rates were observed.

In the groups of patients who received HIPEC, only one case of postoperative intestinal ischemia and one episode of acute renal failure were observed, probably HIPEC-related.

Prognostic factors affecting OS and DFS according to univariate analysis are shown in Table 4.

**Table 4:** Prognostic factors affecting OS and DFS according to univariate analysis in 85 patients with advanced GC

	Overall survival		Disease-free survival	
	%	p*	*n* (%)	p*
Age				
≤ 65	26.1	0.14	20.9	0.23
>65	37.1		31.4	
Gender				
M	26.8	0.23	22.3	0.48
F	38.2		32.5	
ASA score				
≤2	35.7	0.44	27.8	0.23
>2	26.7		22.3	
Tumor location				
Lower third	29.3	0.01	22.4	0.02
Middle/upper third	24.6		19.7	
Neoadjuvant therapy				
Yes	32.8	0.32	27.6	0.25
No	25.7		21.3	
TNM				
<IIIB	33.3	0.02	22.5	0.01
≥IIIB	8		7.7	
PCI**				
<6	31.7	0.01	25.7	<0.0001
≥6	9.3		8.2	
CC score***				
0	30.9	0.009	28.9	<0.0001
>0	9.3		8.6	
R status				
R0	35.7	0.15	29.4	<0.0001
R1/2	26.2		12.9	
Lauren type				
Diffuse	19.5	0.34	13.7	0.25
Others	26.4		22.3	
N. lymph nodes				
<15	27.5	0.67	18.5	0.51
≥15	35.7		21.1	
Positive lymph nodes				
N0	33.6	0.04	29.5	0.05
N+	16.7		15.6	
Type of resection				
Total gastrectomy	21.8	0.01	20.1	0.007
Subtotal distal gastrectomy	36.4		29.3	
Associated resection				
No	30.9	0.16	27.9	0.23
Yes	19.5		17.4	
Operative time, min				
<320	32.1	0.17	26.9	0.2
≥320	21.4		16.7	
Postoperative complications				
Yes	17.6	0.38	15.9	0.66
No	31.7		28.7	
HIPEC				
Yes	30	0.04	25	0.02
No	9		9	
HIPEC				
Prophylactic	33	0.04	30	0.05
Curative	27		20	
Adjuvant therapy				
Yes	31.2	0.71	28.2	0.42
No	26.4		23.6	

*Log-rank test

**PCI Peritoneal Carcinomatosis Index

***CC score cytoreduction completeness score

Tumor location, stage IIIB, PCI ≥6, CC score >0, N+, type of resection, HIPEC, and the type of HIPEC (prophylactic vs curative) significantly affected both OS and DFS. R status significantly affected only DFS (*p* <0.0001).

Table 5 shows multivariate analysis of factors associated with OS and DFS.

**Table 5:** Prognostic factors affecting OS and DFS according to multivariate Cox regression in 85 patients with advanced GC

Variables	OR	95% CI	*p*
5-year OS			
Medium/upper tumor location	1.7	0.95–3.11	0.07
TNM ≥ IIIB	1.49	0.81–2.72	0.19
PCI ≥ 6	1.76	0.77–4.09	0.005
CC ≥0	1.65	0.46–2.43	0.02
N+	1.92	0.73–5.03	0.001
Total gastrectomy	0.73	0.4–1.34	0.32
No HIPEC	1.47	1.23–2.99	0.05
5-year DFS			
Middle/upper third tumor location	1.4	0.78–2.5	0.25
TNM ≥IIIB	1.49	0.83–2.69	0.18
PCI ≥6	2.65	1.23–5.74	0.013
CC ≥0	2.36	0.51–10.92	0.012
R +	2.78	1.18–3.37	0.03
N+	1.75	0.7–4.37	0.22
Total gastrectomy	0.76	0.41–1.41	0.39
No HIPEC	2.52	0.26–1.04	0.005

At the multivariate analysis for OS, PCI ≥6, CC >0, N+ status, and the absence of HIPEC were statistically significant.

On the other hand, at the multivariate analysis, DFS was significantly influenced by PCI ≥6, CC >0, R status, and the absence of HIPEC.

**Fig. 1:**
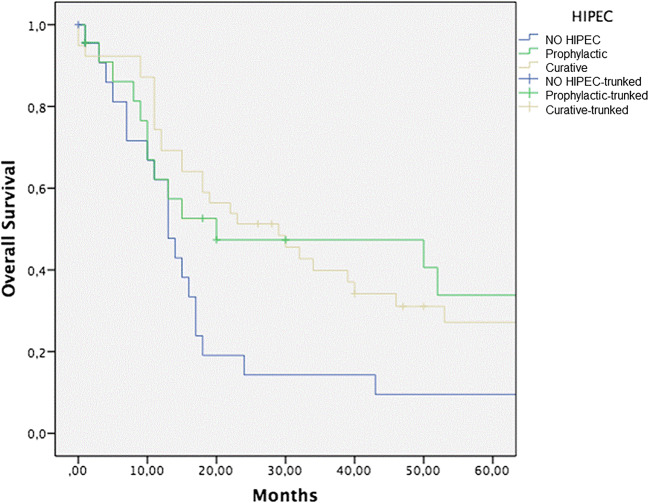
Five-year OS for CRS alone, CRS *plus* curative HIPEC, and CRS *plus* prophylactic HIPEC groups

Five-year OS for surgery alone, CRS + curative HIPEC, and surgery + prophylactic HIPEC groups was 9%, 27%, and 33%, respectively (Fig. 1). Statistical significance was reached comparing both prophylactic HIPEC vs surgery alone group (*p* = 0.05) and curative HIPEC vs surgery alone group (*p* = 0.03).

**Fig. 2:**
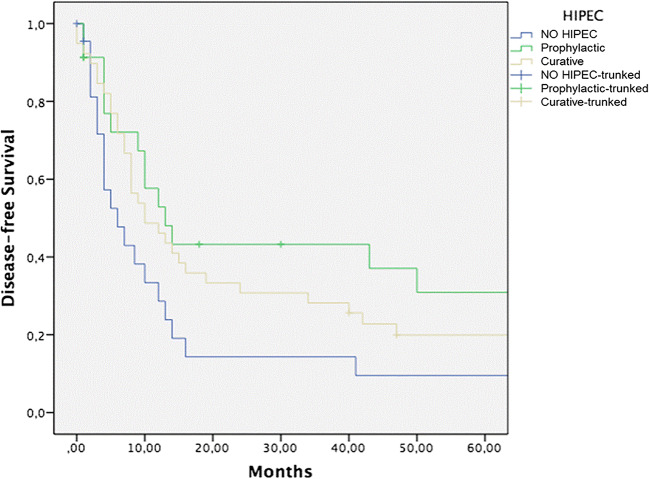
Five-year DFS for CRS alone, CRS *plus* curative HIPEC, and CRS *plus* prophylactic HIPEC groups

Forty-six patients (54.1%) experienced a cancer recurrence, 23 in surgery alone group, 13 in curative HIPEC group, and 10 in prophylactic HIPEC group. In all cases, it was a peritoneal dissemination. Five-year DFS for surgery alone, CRS *plus* curative HIPEC, and surgery *plus* prophylactic HIPEC groups was 9%, 20%, and 30%, respectively (Fig. 2) (*p* = ns). Statistical significance was reached comparing prophylactic HIPEC vs CRS alone group (*p* = 0.008).

The intraperitoneal recurrence rates in patients in surgery *plus* HIPEC with curative intent group, surgery in surgery *plus* HIPEC with prophylactic intent group, and in surgery alone group were 28.2%, 21.7%, and 65.4%, respectively (*p*= 0.007).

## Discussion

Despite high level of evidence, data supporting the use of CRS + HIPEC for treating AGC, with or without PC, it is still not accepted as standard treatment, likely because AGC is still associated with a poor prognosis, even without peritoneal disease [27, 28].

It is well known that intraperitoneal chemotherapy permits a regional drug concentration [17, 29].

Scaringi et al. [30] reported that complete CRS *plus* HIPEC increased advanced AGC patients’ survival rates, especially in those without macroscopic peritoneal residuals.

However, wide application of CRS *plus* HIPEC is hampered by the adverse effects of chemotherapy.

To the best of our knowledge, our study represents the first experience comparing CRS alone, CRS *plus* HIPEC with curative intent, and CRS *plus* HIPEC with prophylactic intent in patients with AGC.

In our paper, we demonstrated that tumor location, advanced T stage, PCI >6, CC score >0, N+, type of resection, and the use of HIPEC significantly affected both OS and DFS. R status significantly affected only DFS (*p* <0.0001). Koga et al. [31] were the first that reported the possible use of HIPEC as a prophylactic treatment for peritoneal recurrence of AGC with serosal invasion. They performed two studies, each with a treated and a control group. In the historical control study, the postoperative 3-year survival rate of patients (73.7%) in the HIPEC group (*n* = 38) was significantly higher than the survival rate (52.7%) of those in the control group (*n* = 55) (*p* < 0.04). In the randomized study, the survival rate (83%) of patients in the HIPEC group (*n* = 26) was also higher than that (67.3%) of those in the control group (*n* = 21) in the 30 months that followed gastric surgery. However, there was no significant difference.

Fujimoto et al. [32], in a prospective study of 59 patients, found that the 30 patients given HIPEC lived longer than the 29 patients not given HIPEC (*p*= 0.001), with a 1-year survival rate of 80.4% in the former group compared to 34.2% in the latter. There have been various randomized controlled trials comparing HIPEC vs no HIPEC in patients with locally AGC who underwent a potentially curative resection [17]. The main limitation of these trials is strictly related to the great heterogeneity with respect to the drugs used, their dosage, duration of HIPEC, temperature achieved, etc. Nevertheless they provide a high level of evidence of the possibility of adjuvant HIPEC to reduce peritoneal recurrence and improve survival.

On the other hand, not many studies have evaluated the effects of prophylactic HIPEC in patients with AGC with positive cytology [33].

Sun et al. [34], in a meta-analysis of ten randomized controlled trials, demonstrated that HIPEC may improve the overall survival rate for patients who receive resection for AGC potentially and help to prevent peritoneal local recurrence among patients with serosal invasion.

In another meta-analysis of 16 randomized controlled trials involving 1906 patients, Mi et al. [28] reported that compared with surgery alone, surgery combined with HIPEC can improve survival rate and reduce the recurrence rate, with acceptable safety.

The GASTRICHIP study is an ongoing prospective, randomized multicenter phase III clinical study with two arms that aims to evaluate the effects of hyperthermic intraperitoneal chemotherapy with oxaliplatin on patients with gastric cancer involving the serosa and/or lymph node involvement and/or with positive cytology at peritoneal washing, treated with perioperative systemic chemotherapy and D1-D2 curative gastrectomy [35]. The most recent meta-analysis by Desiderio et al. [36] demonstrated a survival advantage of the use of HIPEC as a prophylactic strategy and suggests that patients whose disease burden is limited to positive cytology and limited nodal involvement may benefit the most from HIPEC. Moreover, for patients with extensive carcinomatosis, the completeness of cytoreductive surgery is a critical prognostic factor for survival [37]. Future RCTs should better define patient selection criteria.

Sayag-Beaujard et al. [38] reported the first Western experience of extensive surgery *plus* HIPEC. For resectable gastric cancers with stage 1 and 2 carcinomatosis (malignant granulations less than 5 mm in diameter), 1-, 2-, and 3-year survival rates were 80, 61, and 41%, respectively.

In a series by Yonemura et al. [39] on 107 patients who underwent HIPEC, complete cytoreduction was achieved in 47 (43.9 per cent): 18 of 65 who underwent conventional surgery and 29 of 42 who had peritonectomy. Completeness of cytoreduction and peritonectomy were independent prognostic factors. The 5-year survival rate after complete cytoreduction by peritonectomy with HIPEC was 27%.Compared to the most recent literature experiences, our study presented better 5y-OS rates both for curative and prophylactic HIPEC (27% and 33%, respectively) comparing to CRS alone group (9%). Also 5y-DFS rates resulted significantly higher in patients undergoing HIPEC with respect to those who did not (20% and 30% vs 9%, respectively).

The French CYTO-CHIP study by Bonnot et al. [40] is the most recent multicentric study from 19 centers of the FREGAT and the BIG-RENAPE networks that focused especially on the effect of HIPEC after complete CRS using a propensity score analysis. With 277 patients, it represents actually the largest study concerning CRS-HIPEC and gastric cancer. It showed a strong positive effect of HIPEC after CRS versus CRS alone without additional morbidity. Survival rates were similar to those reported in our study. Despite our study represents the first experience comparing HIPEC with curative and prophylactic intent with respect to surgery alone, some major limitations should be evidenced.

First, all data were retrospectively collected, and hence, potential biases could derive from the study design. Second, it reports a single-center non-randomized experience with small sample size groups.

Thirdly, patients with uncontrolled severe infection and/or medical problems unrelated to malignancy were excluded from HIPEC. The selection of treatment results in uncontrollable biases.

Even so, we can conclude that in our experience, in selected patients with AGC, surgery *plus* HIPEC had a better OS and DFS with respect to patients treated with surgery alone.

## Conclusions

In conclusion, according to the results of the present study, patients with AGC undergoing surgery plus HIPEC, both with prophylactic and curative intent, had a better OS and DFS with respect to patients treated with surgery alone. Nevertheless, the role of CRS with HIPEC in AGC with macroscopic PC is still evolving and needs to be addressed in large multi-institutional randomized trials.

Moreover, some issues in the use of HIPEC as an adjuvant treatment in GC—choice of drug, dosage, and duration of treatment— for which there is no consensus are far to be resolved.

Widespread acceptance and adoption of prophylactic and curative HIPEC in AGC requires a satisfactory answer to these issues.

## Declarations

All procedures followed were in accordance with the ethical standards of the responsible committee on human experimentation (institutional and national) and with the Helsinki Declaration of 1964 and later versions.
